# Intervention effect of curcumin on sepsis-associated acute kidney injury via regulation of p300 expression and protein lactylation

**DOI:** 10.1186/s12865-025-00750-3

**Published:** 2025-09-24

**Authors:** Mengyuan Luo, Quanmang Zhu, Guangcai Xu, Dan Liu, Jiajun Xiao, Qiqing Shi

**Affiliations:** 1 Bengbu Clinical Medical Research and Translational Center of Integrated Traditional Chinese and Western Medicine, Bengbu Hospital of Traditional Chinese Medicine, Bengbu, Anhui People’s Republic of China; 2 Department of Anesthesiology, Minhang Hospital, Fudan University, Shanghai, People’s Republic of China; 3 Department of Anesthesiology, Bengbu Hospital of Traditional Chinese Medicine, Bengbu, Anhui People’s Republic of China

**Keywords:** Curcumin, SA-AKI, p300, Protein lactylation, Inflammation

## Abstract

This study investigates the protective effects and underlying mechanisms of curcumin in sepsis-associated acute kidney injury (SA-AKI). Using a cecal ligation and puncture (CLP) model to simulate SA-AKI, our results demonstrate that curcumin significantly reduced serum creatinine and urea nitrogen levels, alleviated tubular damage and inflammation, improved cellular activity, and inhibited apoptosis. Further analysis revealed that curcumin inhibited the expression of p300 and decreased protein lactylation modification in renal tissue, thereby exerting anti-inflammatory and antioxidant effects. These findings suggest that curcumin may have potential therapeutic value for the prevention and treatment of SA-AKI.

**Supplementary Information:**

The online version contains supplementary material available at 10.1186/s12865-025-00750-3.

## Introduction

Sepsis is a systemic inflammatory response syndrome (SIRS) caused by infection, which, when severe, can progress to sepsis-associated acute kidney injury (SA-AKI) [1]. SA-AKI is often accompanied by multiple organ dysfunction and is associated with a significant increase in mortality as well as poor long-term outcomes [2–4]. Therefore, early identification and effective interventions are critical for improving the prognosis of SA-AKI. However, the pathogenesis of SA-AKI is highly complex, and current treatment strategies mainly focus on supportive measures such as antimicrobial therapy, fluid resuscitation, and renal replacement therapy, lacking targeted pharmacological interventions based on its underlying mechanisms [5].

In recent years, traditional Chinese medicine (TCM) has demonstrated unique advantages in alleviating sepsis-induced organ dysfunction. Studies have shown that several active components of TCM, such as Forsythiaside A and Salidroside, can effectively mitigate sepsis-induced organ injury through multiple mechanisms, including anti-inflammatory effects, antioxidative stress, modulation of immune responses, and maintenance of cellular homeostasis [6–8]. Curcumin, a major bioactive compound extracted from Curcuma longa, possesses a wide range of pharmacological activities and has attracted increasing attention for its renoprotective effects in SA-AKI [9]. Curcumin has been shown to ameliorate early renal injury in sepsis by improving renal microcirculatory perfusion and suppressing pro-inflammatory cytokine release. For example, Yang et al. demonstrated that curcumin regulates m6A methylation levels by mediating the transfer of FTO via bone marrow mesenchymal stem cell-derived exosomes, thereby inhibiting the expression of oxidative stress responsive 1 (OXSR1) and attenuating renal inflammation [10]. Additionally, Huang et al. reported that curcumin can downregulate the expression of long non-coding RNA PVT1, inhibit the activation of the JNK/NF-κB signaling pathway, and reduce the production of inflammatory cytokines [11].

Lactylation, a newly identified form of post-translational modification [12], has been increasingly recognized for its role in sepsis-associated acute kidney injury (SA-AKI). Under septic conditions, lactate levels are significantly elevated, accompanied by marked upregulation of the acetyltransferase p300, thereby creating a favorable environment for enhanced lactylation in damaged renal tubular epithelial cells. This modification may exacerbate renal dysfunction by regulating metabolic pathways, inflammatory responses, and apoptosis [13–15]. Previous studies have confirmed that curcumin can inhibit the activity of p300 [16, 17]. Therefore, this study hypothesizes that curcumin may exert its renoprotective effects during the progression of SA-AKI by inhibiting p300 activity and subsequently modulating lactylation levels of relevant proteins.

## Materials and methods

### Animal model and experimental groups

All animal experiments were approved by the Animal Welfare and Ethics Committee of the Laboratory Animal Center, Fudan University (Approval No: 2024-MHYY-33). Male C57BL/6 mice (8–10 weeks old, 20–25 g) were purchased from Zhongshan Hospital, Fudan University (Animal Production License No: SYXK-2016-0006). All animals were housed in a specific pathogen-free (SPF) facility under controlled conditions (temperature 22 ± 2 °C, 12 h light/dark cycle) with free access to food and water for one week of acclimatization prior to experiments.

The SA-AKI model was established using the cecal ligation and puncture (CLP) method. Mice were anesthetized via intraperitoneal injection of sodium pentobarbital (100 mg/kg), and only after confirming a surgical level of anesthesia (complete loss of reflexes and unconsciousness) was a midline laparotomy performed. The distal cecum was then ligated and punctured twice with a sterile needle, and a small amount of fecal content was gently extruded before the cecum was repositioned and the abdominal cavity closed. Sham-operated mice underwent the same surgical exposure but without ligation or puncture. All procedures were conducted in strict accordance with institutional animal welfare guidelines.

After model establishment, eighteen mice were randomly assigned to three groups (*n* = 6 per group): Sham, CLP, and CLP + Curcumin. Mice in the CLP + Curcumin group received curcumin 100 mg/kg (Selleck, USA) by oral gavage at 24 h before CLP surgery and again immediately after the procedure. The curcumin dosage (100 mg/kg) was determined based on preliminary experiments and supported by previous studies [9, 18]. Curcumin was dissolved in corn oil (Sigma, USA). Mice in the CLP and Sham groups received an equivalent volume of corn oil by oral gavage.

At 24 h after CLP surgery, all mice were euthanized under deep anesthesia induced by intraperitoneal injection of sodium pentobarbital (100 mg/kg). Once a surgical level of anesthesia was confirmed, euthanasia was performed by cardiac puncture followed by rapid sacrifice.

### HK-2 cell culture and treatment

Human renal proximal tubular epithelial cells (HK-2, GNHu47) were obtained from the Cell Bank of the Chinese Academy of Sciences (Shanghai, China). Cells were cultured in DMEM/F12 medium supplemented with 10% fetal bovine serum and 1% penicillin-streptomycin. All cultures were maintained in a humidified incubator at 37 °C with 5% CO₂. Cells were seeded into 6-well plates, and when cell confluence reached approximately 70%, they were randomly assigned into three groups for treatment: Control group (Con): treated with an equivalent volume of DMSO as vehicle control; LPS group (LPS): treated with 10 ng/ml lipopolysaccharide (Sigma, USA) dissolved in DMSO for 24 h; LPS + Cur group (LPS + Cur): co-treated with 10 ng/ml LPS and 10 µM curcumin for 24 h. After treatment, cells and medium were collected for subsequent analysis.

### Serum preparation

At 24 h after CLP, mice were euthanized under anesthesia. Blood samples were collected by cardiac puncture and centrifuged at 3000 rpm for 15 min to separate the serum, which was stored at − 20 °C until further analysis.

### Enzyme-Linked Immunosorbent Assay (ELISA)

The levels of IL-1β (Jianglai Bio, China), TNF-α (Jianglai Bio, China), Malondialdehyde (MDA; Jiancheng Bioengineering, China) and lactate (Abcam, UK) in serum or cell culture medium were measured using ELISA kits according to the manufacturers’ instructions. Absorbance was read at 450 nm using a microplate reader (Thermo Fisher Scientific, USA). Standard curves were generated, and data were analyzed accordingly. Each experimental group included six replicate wells.

### Hematoxylin and Eosin (HE) staining of renal tissue

Kidney tissues were fixed in 4% paraformaldehyde, embedded in paraffin, and sectioned at a thickness of 4 μm. Sections were deparaffinized and rehydrated through a graded ethanol series, stained with hematoxylin for 1 min and eosin for 30 s. After dehydration through graded ethanol and clearing in xylene, sections were mounted with neutral resin. Histological changes were observed and photographed under a light microscope.

### Immunohistochemical (IHC) staining of renal tissue

Kidney tissues were fixed in 4% paraformaldehyde, embedded in paraffin, and sectioned at a thickness of 4 μm. Sections were deparaffinized, subjected to antigen retrieval, and blocked with normal serum. Slides were then incubated with primary antibodies overnight at 4 °C. On the following day, sections were incubated with HRP-conjugated secondary antibodies and developed with a chromogenic substrate. Images were captured using a fluorescence microscope.

### CCK-8 assay

HK-2 cells were seeded in 96-well plates at a density of 1*10^6^ cells per well in 100 µL of complete culture medium and incubated at 37 °C with 5% CO_2_. After treatment, Cell Counting Kit-8 (CCK-8; Baoground, China) reagent was added to each well and incubated for an additional 2 h. The optical density (OD) was measured at 450 nm using a microplate reader to evaluate cell viability. Each experimental group included six replicate wells.

### TUNEL assay

HK-2 cells were seeded in multi-well plates and, after treatment, were fixed with 4% paraformaldehyde and permeabilized with 0.1% Triton X-100. The TUNEL assay was performed according to the manufacturer’s instructions (Vazyme, China). Nuclei were counterstained with DAPI, rinsed with PBS, and mounted. TUNEL-positive cells were observed and imaged under a fluorescence microscope to assess the level of apoptosis.

### EP300 overexpression plasmid transfection

For overexpression experiments, HK-2 cells were transfected with EP300 overexpression plasmid (provided by Professor Qiongzhu Dong from the Key Laboratory of Whole-Course Monitoring and Precision Intervention in Digestive Oncology) or empty vector using Lipofectamine™ 3000 Transfection Reagent (Thermo Fisher Scientific, USA) according to the manufacturer’s protocol. After 6 h of transfection, the medium was replaced with fresh complete medium, and cells were cultured for an additional 24 h before being used for downstream assays.

### Western blot analysis

Total protein was extracted from renal tissues or HK-2 cell using RIPA lysis buffer supplemented with a protease inhibitor cocktail. After protein quantification, samples were separated by SDS-PAGE and transferred onto PVDF membranes. Membranes were incubated overnight at 4 °C with Anti-pan lysine lactylation antibodies (Jingjie, China), followed by incubation with appropriate HRP-conjugated secondary antibodies. Protein bands were visualized using an enhanced chemiluminescence (ECL) system. Band intensities were quantified using ImageJ software, and relative protein expression levels were normalized to actin as the internal control.

### Statistical analyses

Statistical analyses were performed using SPSS version 26.0 (IBM, USA) and GraphPad Prism version 8.0 (GraphPad Software, USA). Data normality was assessed using the Shapiro-Wilk test. For normally distributed data, differences between experimental groups were analyzed using one-way analysis of variance (ANOVA). For data not following a normal distribution, the Kruskal-Wallis test was applied. Tukey’s post hoc test was used for multiple comparisons, and a *p*-value < 0.05 was considered statistically significant. In addition to *p*-values, 95% confidence intervals were calculated to provide estimates of the precision and reliability of effect sizes.

## Results

### Curcumin improves renal function and histopathology in septic mice

**Fig. 1:**
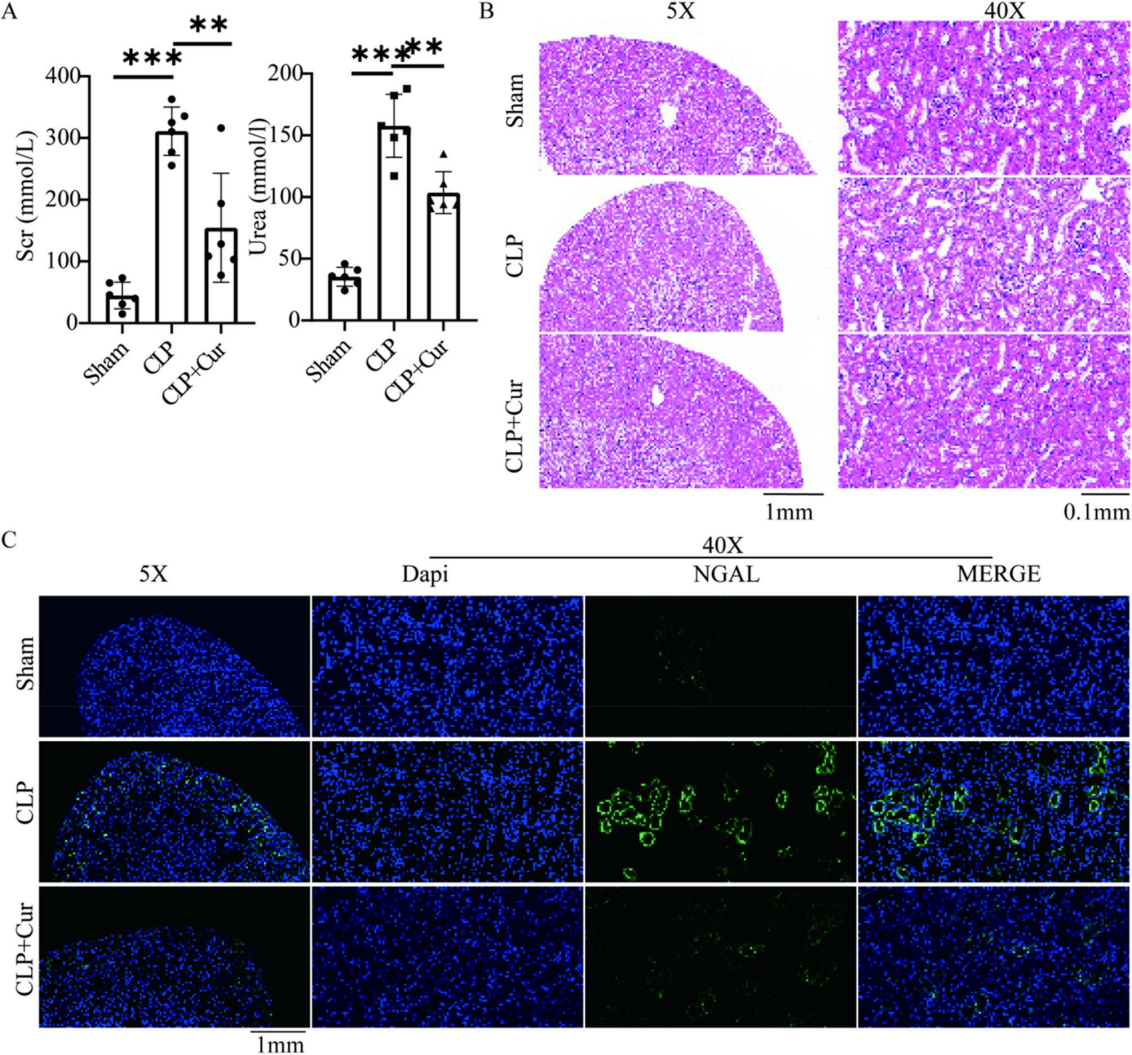
(**A**) Compared with the Sham group, serum creatinine and blood urea nitrogen levels were significantly elevated in the CLP group (*P* < 0.001). These levels were significantly reduced in the CLP + Cur group compared to the CLP group (*P* < 0.001) (**B**) Representative light microscopy images of HE-stained renal tissue. The CLP group exhibited tubular dilation, brush border loss, and flattening and desquamation of tubular epithelial cells compared to the Sham group. The CLP + Cur group showed markedly reduced renal injury compared to the CLP group (**C**) Immunofluorescence images showing NGAL staining. NGAL expression was markedly increased in the CLP group and significantly decreased in the CLP + Cur group

Currently, the diagnosis of AKI is primarily based on elevated serum creatinine levels, with an increase to 1.5 times the baseline considered indicative of AKI occurrence. As shown in Fig. 1A; Table 1, at 24 h post-CLP, serum creatinine (Scr) and blood urea nitrogen (Urea) levels in the CLP group were significantly higher than those in the Sham group [Scr (mean difference: 266.1 mmol/l, 95% CI: 230.2 to 302.0, *P* < 0.001); Urea (mean difference: 122.3 mmol/l, 95% CI: 70.2 to 174.4, *P* < 0.001)], reaching 1.5 times the baseline, indicating successful establishment of the SA-AKI model. Treatment with curcumin (CLP + Cur group) significantly reduced serum creatinine and blood urea nitrogen levels [Scr (mean difference: −156.5 mmol/l, 95% CI: −233.9 to −79.1, *P* < 0.01); Urea (mean difference: −54.1 mmol/l, 95% CI: −78.6 to −29.6, *P* < 0.01)].

**Table 1:** Renal function and inflammation assessment at 24 h Post-CLP in mice

Group (*n* = 6)	Scr (mmol/l)	Urea (mmol/l)	TNF-α (pg/ml)	IL−1β (pg/ml)	Serum Lactate
Sham	44.9 ± 21.6	35.4 ± 7.6	192.0 ± 105.5	1390.6 ± 466.3	1.00 ± 0.37
CLP	311.0 ± 39.2	157.7 ± 25.5	1257.0 ± 312.2	4212.9 ± 676.5	4.02 ± 0.38
CLP + Cur	154.5 ± 88.4	103.6 ± 17.0	359.6 ± 109.7	904.2 ± 369.1	2.76 ± 0.53

Histopathological changes in renal tubular injury were assessed by HE staining and NGAL immunofluorescence. Compared with the Sham group, the CLP group exhibited marked interstitial congestion and edema, increased inflammatory cell infiltration, partial tubular lumen obstruction, and loss of the brush border in tubular epithelial cells. In contrast, the curcumin-treated group (CLP + Cur) showed significantly alleviated tubular structural damage, reduced inflammatory infiltration, and no evident lumen obstruction or brush border loss (Fig. 1B). Furthermore, NGAL-positive staining in renal tubular epithelial cells was significantly lower in the CLP + Cur group than in the CLP group (Fig. 1C). These findings indicate that curcumin markedly improves renal function indices and ameliorates histopathological injury in septic mice.

### Curcumin reduces cytokine and oxidative stress levels, enhances cell viability, and inhibits apoptosis in septic mice

**Fig. 2:**
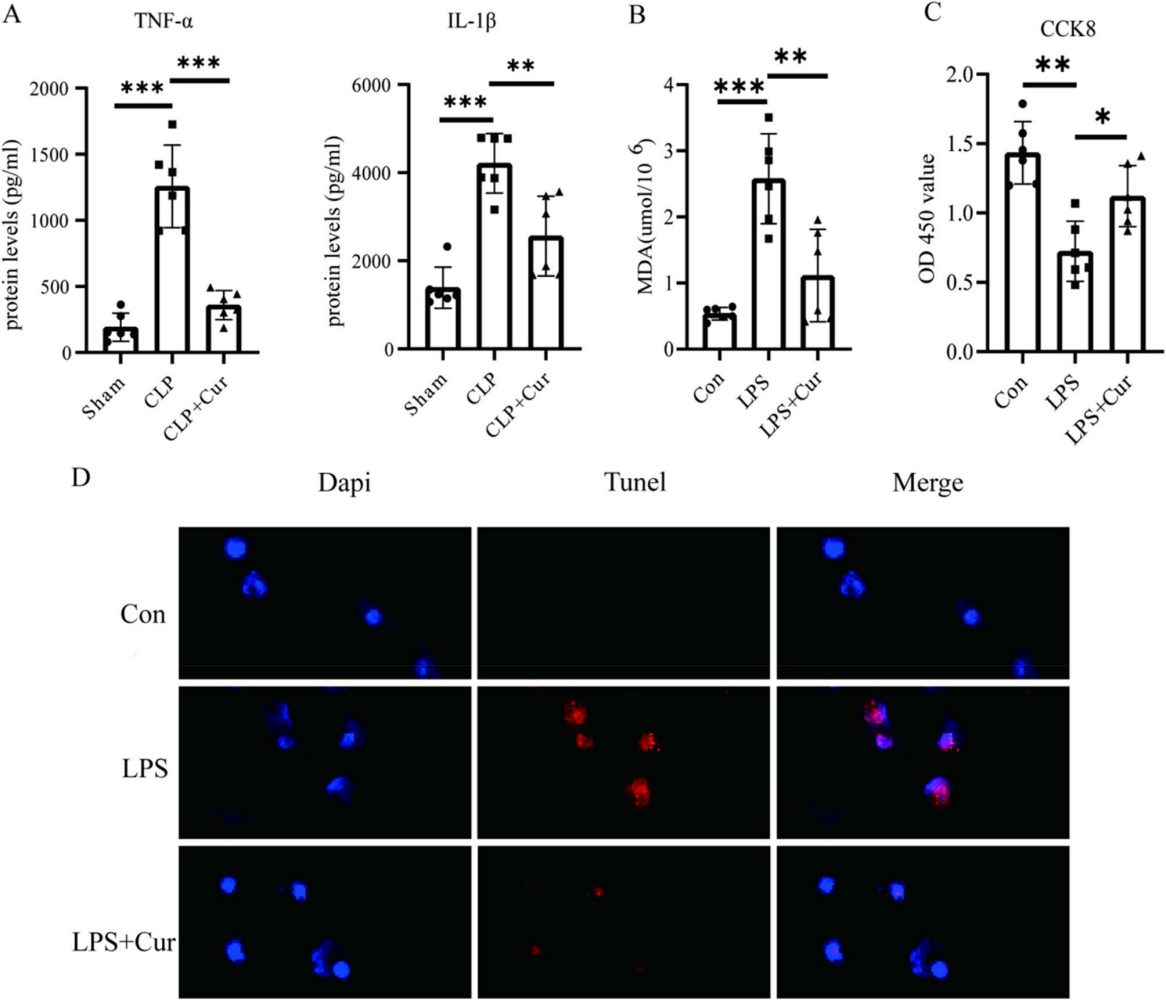
(**A**) Expression levels of TNF-α and IL-1β were significantly increased in the CLP group compared to the Sham group (*P* < 0.001), while curcumin treatment (CLP + Cur group) significantly reduced their expression compared to the CLP group (*P* < 0.001) (**B**) MDA level was significantly increased in the LPS group compared to the Con group (*P* < 0.001) and significantly decreased in the LPS + Cur group compared to the LPS group (*P* < 0.01) (**C**) Cell viability was significantly decreased in the LPS group compared to the Con group (*P* < 0.001) and significantly increased in the LPS + Cur group compared to the LPS group (*P* < 0.001) (**D**) Immunofluorescence images of TUNEL staining. TUNEL-positive signals were markedly increased in the LPS group and significantly decreased in the LPS + Cur group

As shown in Fig. 2A; Table 1, in vivo ELISA results indicated that the levels of inflammatory factors TNF-α and IL-1β in serum were significantly higher in the CLP group than in the Sham group [TNF-α (mean difference: 266.1 pg/ml, 95% CI: 801.4 to 1328.6, *P* < 0.001); IL-1β (mean difference: 2822.3 mmol/l, 95% CI: 2164.9 to 3479.7, *P* < 0.001)]. However, these levels were significantly reduced in the CLP + Cur group [TNF-α (mean difference: −897.4 pg/ml, 95% CI: −1162.2 to −632.6, *P* < 0.01); IL-1β (mean difference: −3308.7 mmol/l, 95% CI: −3925.3 to −2692.0, *P* < 0.01)], suggesting that curcumin may exert its renoprotective effects against SA-AKI by suppressing the inflammatory response.

Further analysis in vitro experiments (Fig. 2B; Table 2) showed that CCK8 assays revealed a significant decrease in renal cell viability (mean difference: −0.71, 95% CI: −0.96 to 0.46, *P* < 0.01) and a marked increase in MDA expression (mean difference: 2.04 µmol/10^6^, 95% CI: 1.49 to 2.59, *P* < 0.001) in the LPS group compared with the Con group. Following curcumin treatment, HK-2 cell viability was significantly increased (mean difference: 0.4, 95% CI: 0.15 to 0.65, *P* < 0.05), while MDA levels were significantly reduced (mean difference: −1.47µmol/10^6^, 95% CI: −2.25 to −0.69, *P* < 0.01), indicating that curcumin helps improve cell viability and mitigate oxidative stress-induced damage.

**Table 2:** Oxidative stress and cellular activity indicators at 24 h Post-LPS in HK-2

Group (*n* = 6)	MDA (µmol/10^6^)	CCK8 (OD 450 value)	Medium Lactate
Sham	0.54 ± 0.09	1.43 ± 0.22	1.0 ± 0.2
LPS	2.58 ± 0.68	0.72 ± 0.22	3.79 ± 1.79
LPS + Cur	1.11 ± 0.70	1.12 ± 0.22	2.24 ± 1.59

As shown in Fig. 2C, TUNEL staining demonstrated that HK-2 cell apoptosis was markedly elevated in the LPS group, whereas curcumin treatment (LPS + Cur group) significantly decreased apoptosis levels. Taken together, these findings suggest that curcumin exerts its renoprotective effects against SA-AKI by lowering renal inflammatory cytokine levels and oxidative stress, enhancing cellular viability, and inhibiting apoptosis.

### Curcumin reduces lactate levels in vivo and vitro experiments

**Fig. 3:**
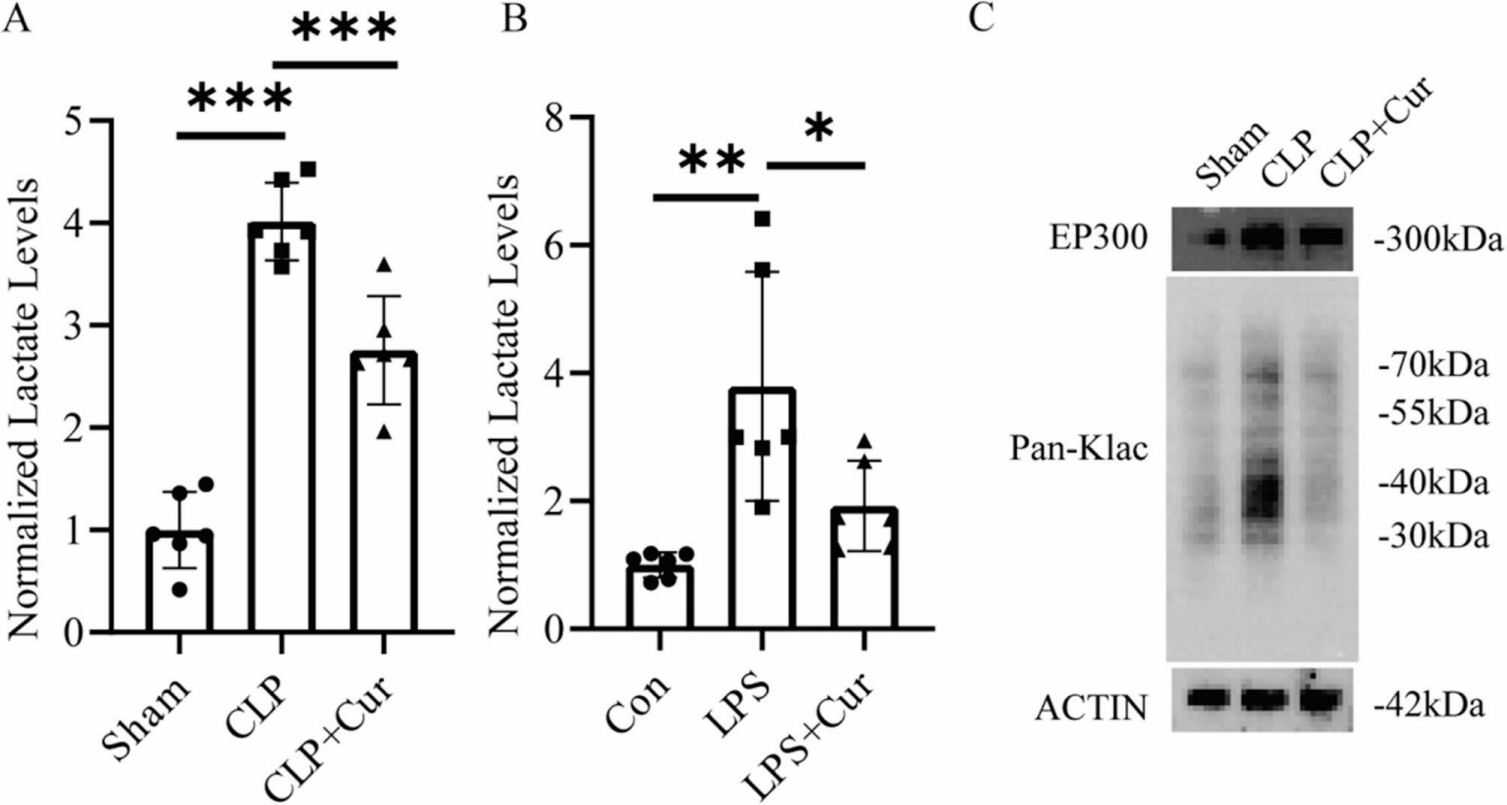
(**A**) In vivo, serum lactate levels were significantly increased in the CLP group compared to the Sham group (*P* < 0.001). These levels were significantly decreased in the CLP + Cur group compared to the CLP group (*P* < 0.001) (**B**) In vitro, cell culture medium lactate levels were significantly increased in the LPS group compared to the Con group (*P* < 0.01). These levels were significantly decreased in the LPS + Cur group compared to the LPS group (*P* < 0.05) (**C**) Total protein lactylation levels in renal tissues were markedly elevated in the CLP group compared to the Sham group and were significantly reduced in the CLP + Cur group compared to the CLP group

Sepsis activates anaerobic metabolism and suppresses aerobic metabolism, leading to increased lactate production. Previous studies have shown that serum lactate levels are closely correlated with the severity of SA-AKI. As shown in Fig. 3A; Table 1, in vivo experiments ELISA results indicated that serum lactate concentrations were significantly higher in the CLP group than in the Sham group (mean difference: 3.02, 95% CI: 2.59 to 3.45, *P* < 0.001). Curcumin treatment (CLP + Cur group) significantly reduced serum lactate levels compared to the CLP group (mean difference: −1.26, 95% CI: −1.79 to −0.73, *P* < 0.001). Similarly, in vitro experiments, as shown in Fig. 3B; Table 2, lactate concentrations in the cell culture supernatant were markedly elevated in the LPS group compared to the Con group (mean difference: 2.79, 95% CI: 1.34 to 4.24, *P* < 0.01). Notably, curcumin intervention (LPS + Cur group) significantly decreased lactate levels (mean difference: −1.55, 95% CI: −3.45 to 0.35, *P* < 0.05).

Lactate serves as a key substrate for protein lactylation; thus, elevated lactate concentrations inevitably enhance protein lactylation levels within renal tissue. Previous reports have demonstrated that protein lactylation plays a crucial role in the pathogenesis of SA-AKI. As shown in Fig. 3C, the level of protein lactylation in renal tissue was significantly increased in the CLP group compared to the Sham group, whereas curcumin administration (CLP + Cur group) markedly reduced the degree of lactylation. These findings suggest that curcumin may exert its renoprotective effects against SA-AKI by attenuating lactate accumulation and the consequent lactylation modifications.

### Curcumin exerts renoprotective effects in SA-AKI by suppressing p300 expression and protein lactylation

p300 is an acetyltransferase that also facilitates protein lactylation; its upregulation promotes increased lactylation levels. It has been reported that p300 expression is significantly elevated under septic conditions. As shown in Fig. 3C, Western blot analysis revealed that renal p300 expression was markedly higher in the CLP group than in the Sham group, while curcumin treatment (CLP + Cur group) significantly reduced p300 expression levels. These results indicate that the renoprotective effects of curcumin in SA-AKI may be mediated by inhibition of p300 expression.

**Fig. 4:**
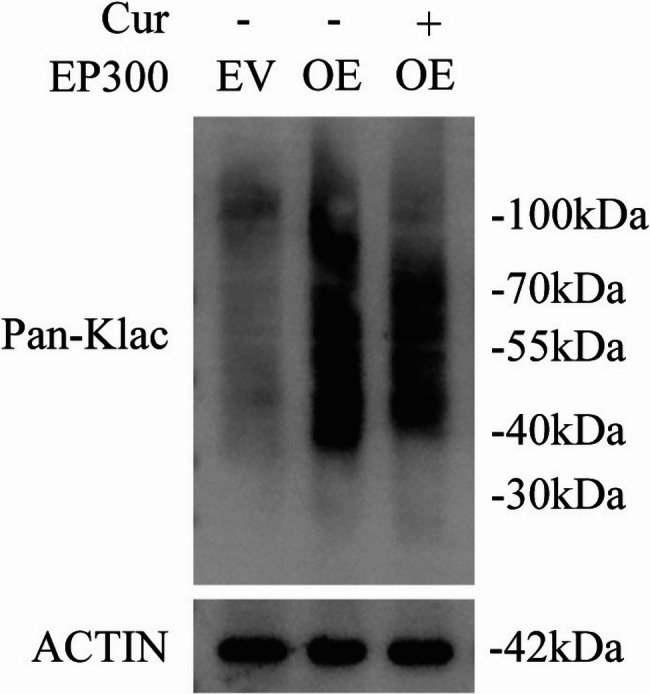
Compared with HK-2 cells transfected with an empty vector, those transfected with an EP300 overexpression plasmid exhibited a significant increase in total protein lactylation levels, which were notably reduced following additional treatment with curcumin

To further validate this mechanism, an HK-2 cell model was employed. Cells were transfected with an EP300 expression plasmid or an empty vector, with some cells pre-treated with curcumin. As shown in Fig. 4B, Western blot results demonstrated that overexpression of p300 significantly increased cellular protein lactylation levels, and under this condition, curcumin could no longer reduce lactylation. These findings further confirm that the renoprotective effect of curcumin depends on its ability to suppress p300 expression, thereby attenuating activation of lactylation-associated pathways and exerting comprehensive anti-inflammatory and antioxidant protective effects.

## Discussion

Sepsis is one of the leading causes of acute kidney injury (AKI), which is typically characterized by a significant increase in renal function indicators such as serum creatinine and blood urea nitrogen, as well as pathological alterations in renal tissue structure. Sepsis can also induce severe oxidative stress in the kidneys, as evidenced by elevated levels of MDA, a key end product of lipid peroxidation, and is accompanied by marked upregulation of inflammatory cytokines including IL-1β and TNF-α. In this study, we successfully established a sepsis mouse model using the CLP method and observed that curcumin exerted a significant renoprotective effect in this model.

Curcumin, a natural polyphenolic compound, is widely recognized for its potent antioxidant and anti-inflammatory properties. In this study, curcumin administration significantly decreased elevated renal function indicators, mitigated histopathological renal damage, and improved oxidative stress and inflammatory responses, underscoring its therapeutic potential in preventing nephrotoxic injury. In vitro, using the LPS-induced inflammatory model of HK-2 cells, we confirmed that curcumin alleviates cellular injury and suppresses inflammatory responses. Mechanistically, this effect is likely mediated through inhibition of the JAK2/STAT3 and NF-κB signaling pathways, which are central regulators of LPS-induced cytokine production, including IL-6 and TNF-α [18]. Our findings are consistent with prior research highlighting the fundamental role of oxidative stress and inflammation in renal injury. Notably, natural compounds such as tyrosol and carvacrol have demonstrated similar renoprotective effects by attenuating these pathological pathways in diverse nephrotoxicity models [19, 20]. These converging lines of evidence suggest that targeting oxidative and inflammatory mechanisms represents a broadly applicable and clinically relevant strategy for renal protection.

Recent studies have demonstrated that lactate plays a crucial role in the onset and progression of SA-AKI and is considered an important independent predictor of AKI. Lactate is a metabolic byproduct of cellular glycolysis and serves as a sensitive indicator for evaluating systemic perfusion status. During sepsis, impaired tissue perfusion leads to excessive lactate production and its release into the bloodstream, resulting in hyperlactatemia. Clinical evidence indicates that patients with hyperlactatemia generally exhibit higher APACHE II scores, an increased incidence of shock and multiple organ dysfunction syndrome (MODS), and significantly higher mortality rates. Specifically, when blood lactate levels are ≥ 4 mmol/L, the mortality rate in septic patients increases markedly [21]. Additionally, a meta-analysis by Liu et al. reported that lactic acidosis, diabetes, a mean arterial pressure (MAP) < 65 mmHg, and coagulation disorders are significant risk factors for SA-AKI [22]. At the cellular level, Tan et al. found that LPS stimulation of HK-2 cells significantly upregulated the expression of glycolysis-related genes, thereby promoting lactate production. The accumulated lactate further suppressed the expression of SIRT3 and phosphorylated AMPK (p-AMPK), resulting in reduced autophagy and increased apoptosis [23]. Collectively, these findings suggest that lactate is not only an important biomarker for the occurrence and development of SA-AKI but also actively participates in its pathogenesis at the molecular level. This is further supported by recent findings from metabolic profiling studies, which revealed that sepsis-induced AKI is associated with profound metabolic reprogramming, particularly involving lactate-driven oxidative stress and amino acid dysregulation. These changes contribute to renal energy imbalance and inflammation, exacerbating organ dysfunction [24].

Recent mechanistic studies have demonstrated that lactate plays an active role in the pathogenesis of SA-AKI by promoting protein lactylation. Notably, lactate can induce the lactylation of high mobility group box 1 (HMGB1), a key damage-associated molecular pattern (DAMP) involved in inflammatory signaling [25]. This modification enhances the release of neutrophil extracellular traps (NETs), DNA-protein complexes implicated in inflammation and thrombosis, thereby exacerbating renal injury. A positive correlation between plasma NETs levels and HMGB1 lactylation further supports the existence of a lactate–HMGB1–NETs axis in SA-AKI progression. In parallel, histone lactylation has emerged as another important epigenetic mechanism contributing to SA-AKI. Specifically, elevated levels of H3K18 lactylation (H3K18la) have been observed in SA-AKI mouse models. CUT&Tag analysis revealed that H3K18la is enriched in promoter regions, particularly in renal tubular epithelial cells, with downstream genes enriched in pathways related to cytoskeletal remodeling, cellular metabolism, and inflammatory responses. Functionally, H3K18la has been shown to enhance NF-κB pathway activation and aggravate inflammation, whereas its suppression alleviates renal dysfunction [14]. These findings align with growing evidence that epigenetic mechanisms—such as histone modifications, DNA methylation, and noncoding RNAs—play pivotal roles in regulating inflammatory and metabolic pathways in SA-AKI, offering promising targets for diagnosis and therapy [26]. In our study, curcumin treatment was associated with reduced global lactylation levels in renal tissue. However, the specific downstream substrates modulated by curcumin remain unidentified. Further investigations using proteomics or mass spectrometry approaches are needed to determine whether curcumin directly affects the lactylation of HMGB1, H3K18, or other key proteins.

As a key acetyltransferase, p300 not only participates in histone acetylation but also plays a crucial role in protein lactylation. Previous studies have demonstrated that p300, through its catalytic activity, can promote the lactylation modification of various substrate proteins, including histones and inflammation-related signaling molecules, thereby regulating cellular processes such as inflammatory responses, apoptosis, and oxidative stress. Elevated expression of p300 has been consistently observed in sepsis and associated organ injuries, suggesting its central role in the pathogenesis of SA-AKI [27]. Consistent with these findings, our study showed that curcumin significantly downregulated p300 expression, thereby markedly suppressing protein lactylation levels in renal tissue, which in turn alleviated oxidative stress and inflammatory responses in the kidney. This not only highlights the pivotal role of p300 in the pathophysiology of SA-AKI but also suggests that p300 may serve as an important regulator of lactylation levels and their pathological consequences. Future investigations could focus on the therapeutic potential of selective p300 inhibitors or p300 gene silencing strategies in SA-AKI, as well as elucidating the specific substrate spectrum and functional roles of p300-mediated lactylation. Such research would expand our understanding of lactylation and p300-related signaling pathways, providing a more targeted approach for the treatment of SA-AKI and other inflammation-related diseases.

This study still has certain limitations: although we observed that curcumin reduces the overall level of protein lactylation in SA-AKI, the specific lactylated substrate proteins and their downstream functional pathways have not yet been fully characterized. In particular, whether curcumin directly modulates the lactylation of key nuclear or inflammatory proteins, such as HMGB1 or histone H3K18, remains unclear. Future studies employing targeted validation or proteomics-based approaches (e.g., mass spectrometry) are warranted to identify these specific targets and elucidate their roles in the pathogenesis of SA-AKI, thereby providing a more robust foundation for clinical translation. In addition, it is worth noting that curcumin exhibits inherently poor oral bioavailability, which may limit its translational application. Although the dose and regimen used in this study were based on prior literature and validated by pre-experiments, no bioavailability enhancers were applied. Future research should consider the use of delivery strategies or adjuvants (e.g., piperine, nanoparticles, or liposomes) to improve the systemic absorption and therapeutic efficacy of curcumin.

## Conclusion

In summary, our findings indicate that curcumin exerts significant renoprotective effects by inhibiting protein lactylation modifications in renal tissue, demonstrating its potential to alleviate sepsis-associated acute kidney injury. This provides novel insights and potential therapeutic targets for the intervention and treatment of SA-AKI.

## Supplementary Information

Supplementary Material 1. Supplementary Figure: full-length Western blots of Figs. 3 C and 4.

## Data Availability

Data and material are available on request.

## Abbreviations

SA-AKI: Sepsis-associated Acute Kidney Injury
CLP: Cecal Ligation and Puncture
HK-2: Human proximal tubular epithelial cells
MDA: Malondialdehyde
