# Supplementary figures and images for: Intervention effect of curcumin on sepsis-associated acute kidney injury via regulation of p300 expression and protein lactylation
Source: BMC Immunol. 2025 Sep 24;26:67. doi: 10.1186/s12865-025-00750-3 (PMC12459039; doi:10.1186/s12865-025-00750-3)

FIG-3C

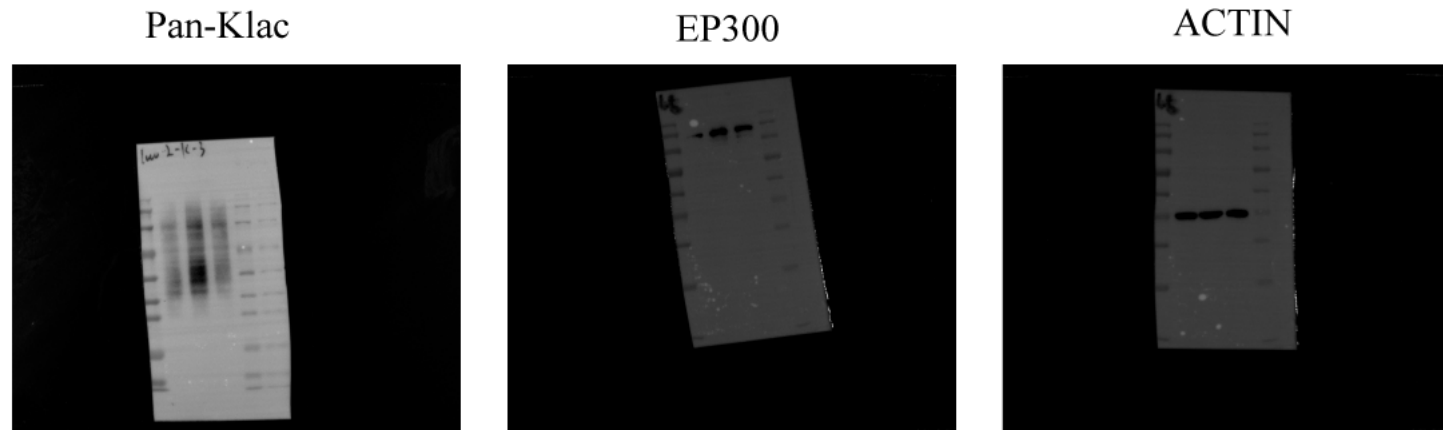

FIG-4

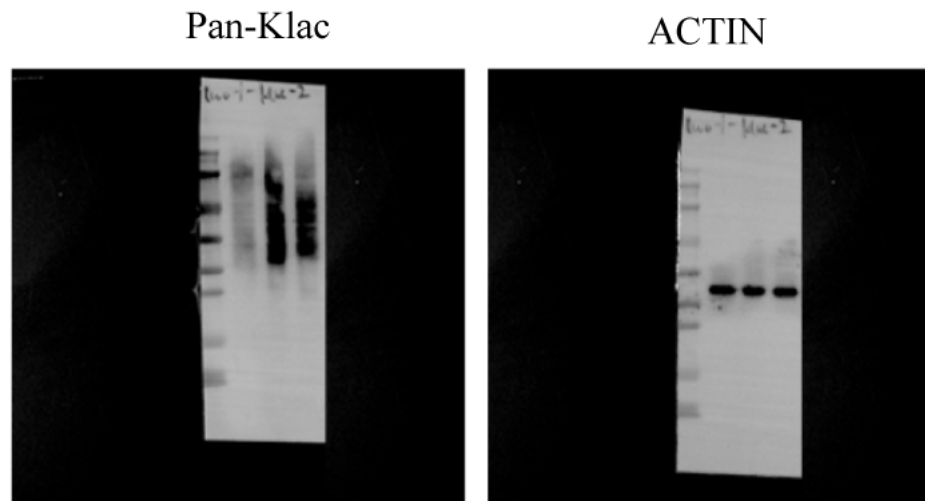

Supplement: Supplementary file 1 — Supplementary Material 1. Supplementary Figure: full-length Western blots of Figs. 3 C and 4. [file 12865_2025_750_MOESM1_ESM.pdf]
